# Application of a population-based severity scoring system to individual patients results in frequent misclassification

**DOI:** 10.1186/cc3790

**Published:** 2005-08-09

**Authors:** Frank V Booth, Mary Short, Andrew F Shorr, Nancy Arkins, Becky Bates, Rebecca L Qualy, Howard Levy

**Affiliations:** 1 Medical Fellow, Eli Lilly and Company, Indianapolis, IN, USA; 2 Associate Clinical Research Scientist, Eli Lilly and Company, Indianapolis, IN, USA; 3 Associate Director of Pulmonary Critical Care Medicine, Pulmonary and Critical Care Medicine, Washington Hospital Center, Washington, DC, USA and Associate Professor of Medicine, Georgetown University, Washington, DC, USA; 4 Senior Clinical Development Associate, Eli Lilly and Company, Indianapolis, IN, USA; 5 Associate Senior Statistician, Eli Lilly and Company, Indianapolis, IN, USA; 6 Senior Scientific Communication Associate, Eli Lilly and Company, Indianapolis, IN, USA; 7 Medical Director, Eli Lilly and Company, Indianapolis, IN, USA+

## Abstract

**Introduction:**

APACHE II (AP2) was developed to allow a systematic examination of intensive care unit outcomes in a risk adjusted manner. AP2 has been widely adopted in clinical trials to assure broad consistency amongst different groups. Although errors in calculating the true AP2 score may not be reducible below 15%, the self-canceling effect of random errors reduces the importance of such errors when applied to large populations. It has been suggested that a threshold AP2 score be used in clinical decision making for individual patients. This study reports the AP2 scoring errors of researchers involved in a large sepsis trial and models the consequences of such an error rate for individual severe sepsis patients.

**Methods:**

Fifty-six researchers with explicit training in data abstraction and completion of the AP2 score received scenarios consisting of composites of real patient histories. Descriptive statistics were calculated for each scenario. The standard deviations were calculated compared with an adjudicated score. Intraclass correlations for inter-observer reliability were performed using Shrout-Fleiss methodology. Theoretical distribution curves were calculated for a broad range of AP2 scores using standard deviations of 6, 9 and 12. For each curve, the misclassification rate was determined using an AP2 score cut-off of ≥25. The percentage of misclassifications for each true AP2 score was then applied to the corresponding AP2 score obtained from the PROGRESS severe sepsis registry.

**Results:**

The error rate for the total AP2 score was 86% (individual variables were in the range 10% to 87%). Intraclass correlation for the inter-observer reliability was 0.51. Of the patients from the PROGRESS registry. 50% had AP2 scores in the range 17 to 28. Within this interquartile range, 70% to 85% of all misclassified patients would reside.

**Conclusion:**

It is more likely that an individual patient will be scored incorrectly than correctly. The data obtained from the scenarios indicated that as the true AP2 score approached an arbitrary cut-off point of 25, the observed misclassification rate increased. Integrating our study of AP2 score errors with the published literature leads us to conclude that the AP2 is an inappropriate sole tool for resource allocation decisions for individual patients.

## Introduction

The Acute Physiology and Chronic Health Evaluation II (APACHE II) scoring system was originally developed as a tool for comparing the outcomes of acute disease in critically ill patients across multiple intensive care units in a therapy-independent fashion [1]. Although relatively few critical care units have adopted this system or its successor, APACHE III, for this purpose, APACHE II has found widespread application in clinical trials as a tool both for stratification of patient populations and as a means of demonstrating acceptable baseline balance amongst subgroups within a given trial. In large groups of patients, it has repeatedly been demonstrated that there is excellent correlation between APACHE II score and risk of death. The actual mortality risk predicted by this scoring system varies considerably with the underlying diagnosis and from country to country. The developers of APACHE II have emphasized that an accurate classification of the underlying disease state is essential for the accuracy of the predictive model [1].

The total APACHE II score is derived by summing points from three distinct categories: acute physiologic derangements (12 individual elements); age points; and points for the presence of certain specific chronic health conditions or medical situations. Within the acute physiologic score, three elements require additional decisions or preparatory calculation: the Glasgow coma score; an assessment of pulmonary function; and a decision if an abnormal value of creatinine represents acute or chronic renal failure. The difficulties of reliably determining Glasgow Coma Score have been well documented. In assessing pulmonary function, depending on the fraction of inspired oxygen (F_i_O_2_), either the arterial partial oxygen pressure (pO_2_) or the alveolar-arterial oxygen gradient (A-a DO_2_) must be used. The calculation of the latter requires the successful application of the alveolar gas equation, which in turn requires knowledge of average local atmospheric pressure. These numerous and complex data manipulations required to calculate the APACHE II score introduce many opportunities for error in the determination of an individual patient's points total. The combination of many elements into a composite score means that there are literally thousands of data permutations, which may be recorded to produce an identical APACHE II score.

This retrospective study reports the APACHE II scoring error rates for three case scenarios calculated by Clinical Research Associates and Research Coordinators involved in a large randomized placebo-controlled critical care clinical trial. We examined the effects of these scoring error rates on the ability to correctly classify an individual into either having an APACHE II score above or below a cut-off score of 25. In addition, we used a large database of patients with severe sepsis to estimate the distribution of reported APACHE II scores. Combining this known distribution of APACHE II scores and our estimated misclassification rates, we estimated the overall frequency of misclassification of individual severe sepsis patients into categories of having an APACHE II score above or below 25.

## Methods

### Study participants

Fifty-six individuals (clinical research associates (n = 17) and study coordinators (n = 39), associated with the ADDRESS clinical trial) returned completed case scenarios used in this study. Demographic data on these individuals were not obtained. All received explicit training in data abstraction and recording for the ADDRESS trial, a multi-institutional investigation of drotrecogin alfa (activated) in severe sepsis. Study procedures for this trial required that APACHE II score be obtained at baseline, either from the medical record if this calculation was part of the clinical routine at the specific institution or as a study-specific determination. The study coordinators came from individual participant sites in the ADDRESS trial and were either employees or associates of the principal investigators at those sites. The clinical research associates were either employees of Eli Lilly and Company or of a contract research organization engaged by Lilly to assist in the conduct of the ADDRESS trial. The case scenarios, instructions and scoring sheets for APACHE II were distributed to the participants at the beginning of a two-day study initiation meeting and were returned at its conclusion. Participants completed these forms individually. No constraints were applied on the time allowed for completion. Participants were given the option of returning the score sheets anonymously or with their names included (for the purpose of receiving feedback). Almost without exception, score sheets were returned bearing the participant's name, but were subsequently obliterated and replaced with an anonymous identifier for the purposes of data analysis for this study.

### Case scenarios

Three individual case scenarios were developed using composites of real patient histories and laboratory values. Each scenario consisted of several elements but all contained at a minimum: a multi-page critical-care vital signs flow sheet (with multiple and frequent observations of pulse rate, blood pressure, respiratory rate, components of the Glasgow coma score, etc.); and a laboratory values report in the form of a spreadsheet, typically covering a 48 h period and including 18 routine chemistries, cardiac enzymes, arterial and venous blood gas values as well as routine hematology results. The third element of the scenario was a narrative summary of the patient's clinical course. In many cases this summary contained items of relevance to the calculation of an APACHE II score, such as times of landmark events, and physiologic values observed in the pre-hospital or emergency room environment. The participants were given a standardized APACHE II scoring sheet and instruction set.

### Adjudicated APACHE II score

Two of the authors (MS and FVMcLB) independently scored each clinical scenario on two separate occasions approximately two weeks apart. A consensus-forming session was then held at which every individual contributing element of the APACHE II score was reviewed, agreed upon and an adjudicated point value determined. For one of the scenarios (APACHE II score = 22) the agreed aggregate point value was identical to the value calculated by the two observers independently. For the other two, an adjustment of a single point was agreed upon. These consensus values were then used as the adjudicated values against which the scores of the study participants were measured.

### Statistical methodology

Descriptive statistics (mean, median, inter-quartile range) were calculated for each scenario. The standard deviations were calculated using the adjudicated APACHE II value in place of the mean reported APACHE II score.

Intraclass correlations for inter-observer reliability were performed using Shrout-Fleiss methodology [2]. The intraclass correlation used in this study assumed the same observers scored the three scenarios, although each scenario was a random subset of all possible observers. In the second phase of this study, it was assumed that for any given population of patients with an identical true APACHE II score, the distribution of possible APACHE II scores would be approximately normal. Theoretical distribution curves were calculated for each true APACHE II score using standard deviations of 6, 9 and 12. For each distribution curve, the misclassification rate was determined in the following manner. If the true score was <25, misclassification was represented by the area of the distribution curve above or equal to 25. If the true score was ≥25, misclassification was represented by the area of the distribution curve below 25.

A large sample of APACHE II scores (n = 5,253) was obtained from the PROGRESS registry, a collaborative web-based registry of severe sepsis patients admitted to over sixty intensive care units worldwide [3]. The percentage of misclassifications for each true APACHE II score estimated in the second phase of this study was applied to the corresponding scores in this large sample of APACHE II scores. An overall misclassification rate was estimated by summing the misclassifications for each APACHE II score from this sample.

## Results

Not every participant completed every case scenario; the completion rate was 159/168 (94.6%). Fifteen participants returned composite scores only. The three different scenarios had widely differing adjudicated APACHE II scores. The scenario with an adjudicated score of 44 was most frequently scored incorrectly (52/56, 92.9% incorrect). The accuracy of scoring was better for the other two scenarios whose adjudicated scores were markedly lower (score = 22: 45/52, 86.5 % incorrect; score = 19: 41/43, 77.4% incorrect). In only two of the numerically correct total scores did the participant arrive at their answers by a balanced combination of errors.

**Figure 1:**
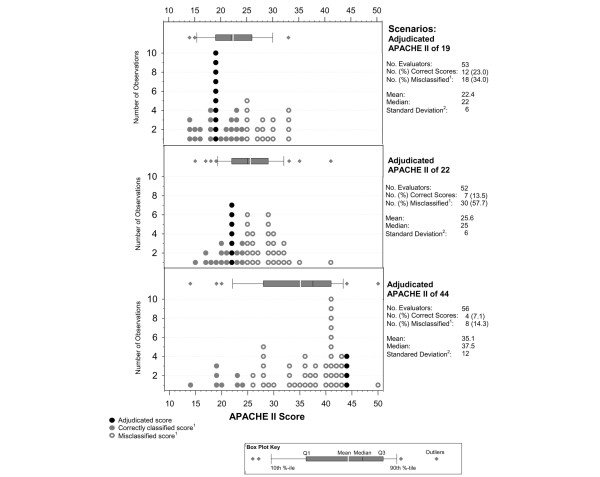
Results of the scoring exercise. The results of the scoring exercise completed by researchers involved in a large randomized placebo-controlled critical care trial illustrating individual scores, standard deviations and inter-quartile ranges of case scenarios with adjudicated total APACHE II scores of 44, 22 and 19. ^1^Correct classification is determined by the adjudicated score being either APACHE II ≥25 or APACHE II <25. ^2^Standard deviation is calculated using the adjudicated APACHE II score in place of the mean APACHE II score.

In contrast to the scenario with a score of 44 in which all but one of the erroneous scores underestimated the true APACHE II score, the distribution of the erroneous scores assumed a more normal random distribution for scenarios with scores of 19 and 22. The intraclass correlation for the inter-observer reliability was 0.51, 95% CI (0.22–0.98). The results of the scoring exercise, individual scores, standard deviations and interquartile ranges are shown in Fig. 1.

**Figure 2:**
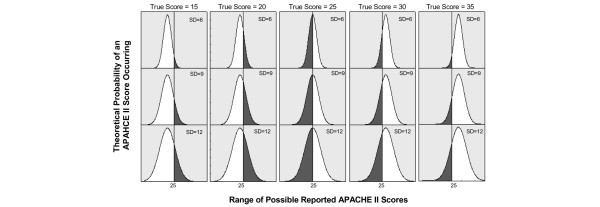
Theoretical distributions of APACHE II scores with varying SDs. A set of theoretical distributions of reported APACHE II scores based on standard deviations of 6 and 12 (which were what we observed in the case scenario data.) For the purposes of comparison, a set of curves using an intermediate standard deviation of 9 is also shown. In every curve, the shaded area illustrates the theoretical probability of misclassification based on a cut-off score of ≥25.

Table 1 lists the error rate for each component of the APACHE II score. Fig. 2 shows the theoretical distribution curves of five true values of APACHE II scores. The areas shaded show the proportion of scores that would result in a misclassification using an APACHE II score cut-off of 25 or greater. The value of 25 was chosen because it has been suggested that this value may be used to identify a patient at high risk of death from severe sepsis. The effect of varying the assumed standard deviation is also shown. The proportion of misclassification increases as the true score approaches the cut-off score of 25. The highest rate of misclassification occurs when the true score equals the cut-off score.

**Figure 3:**
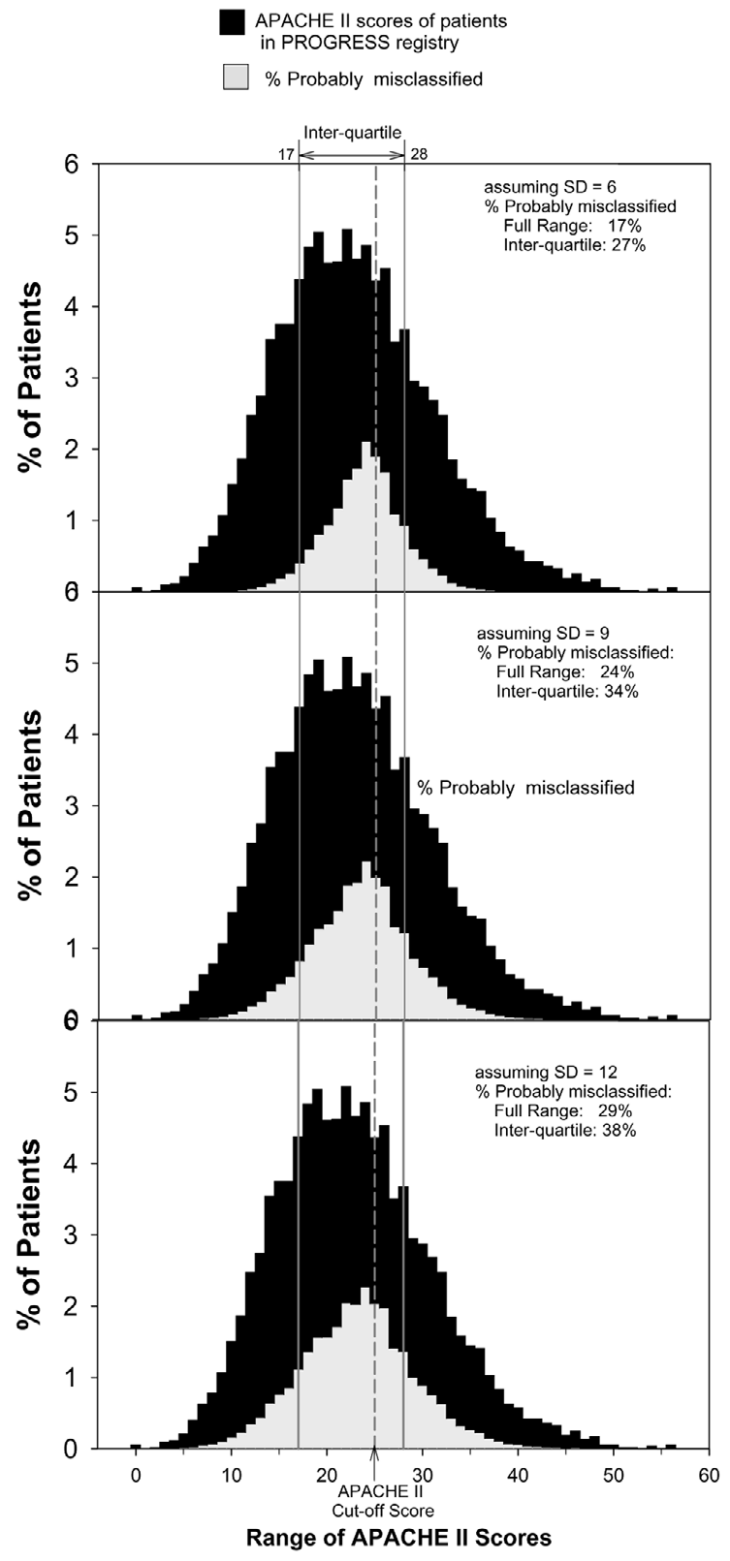
Distribution of reported APACHE II scores in the PROGRESS registry. The darker shading (outer envelope) of these plots represents the observed distribution of APACHE II scores of 5,253 severe sepsis patients in the PROGRESS registry. The lighter shading (inner envelope) is calculated by applying the probability of misclassification for each individual APACHE II score based on assumed standard deviation (SD) of (from top to bottom) 6, 9 and 12 and on an APACHE II cut-off score ≥25.

Fig. 3 shows the relative frequency of APACHE II scores observed in a population of severe sepsis patients (PROGRESS Registry). The lightly shaded areas in Fig. 3 show the estimated distribution of misclassification rates of individuals with severe sepsis into groups of scores <25 and ≥25 based on the estimated misclassification rates from the theoretical distribution curves. Using this distribution of APACHE II scores from the PROGRESS registry, 50% of severe sepsis patients have APACHE II scores ranging from 17 to 28. Within this interquartile range will reside 70% to 85% of all misclassified patients (depending on the underlying standard deviation of the APACHE II scores).

## Discussion

Our observations concerning the accuracy and inter-observer variability of deriving APACHE II scores in a simulated clinical setting are concordant with those of Polderman *et al*. [4] and Chen *et al*. [5], although the absolute magnitude of the errors we report is larger. Fig. 1 shows the differing distributions of erroneously calculated scores. The scenario with a score of 44 has a mean and median that substantially underestimate the adjudicated APACHE II score, whereas the distribution of errors in the other two scenarios resulted in a mean and median within three APACHE II points of the adjudicated score. This would be considered statistically indistinguishable from the adjudicated value and acceptable from a scientific sampling point of view when comparing population intensive care unit outcomes, or the success of randomizing patients into subgroups with comparable severity.

Furthermore, it should be noted that in our study, Glasgow coma scores as recorded in the flow sheets were assumed to be accurate. In nursing practice, errors in reporting of Glasgow coma scores, especially for the intubated patient, are well documented [6]. APACHE II methodology requires that scores for creatinine intervals be doubled for acute renal failure. Overall, creatinine points were assessed incorrectly 43% of the time. In one of the scenarios, however, all the creatinine values were within normal limits. Thus, on the score sheets where the creatinine score should have been doubled, this step was omitted on 64 of a possible 72 (89%) occasions, making omission of this step in practice the most frequent error seen.

In this simulation the practitioners were provided with a summary clinical abstract, which was assumed to contain all the relevant clinical and time-line information. In the clinical environment such information is frequently intimately interwoven with extraneous confusing and irrelevant matter within a complex clinical chart. This likely increases the possibility that an important point of information may be overlooked. Table 1 demonstrates that even invariable information such as age was incorrectly abstracted or assigned to the incorrect interval that determines the score for that parameter, and that for many parameters an incorrect value was more likely to be assigned than a correct value. In selecting the range of standard deviation used for the illustrative theoretical curves (Fig. 2), we relied on values observed in the case scenarios (standard deviations of 6 and 12), and arbitrarily included a standard deviation of 9.

In reviewing the technical literature of APACHE II, two distinct approaches are seen: the overall performance of the score as an outcome predictor for groups of patients; and the performance of small groups of individuals in achieving accuracy and reproducibility of the actual APACHE II score. With regard to the much more frequent reporting of overall performance of the score as an outcome predictor for groups of patients, all of these types of studies have the underlying assumption that there is a sufficiently large patient sample size to ensure that any effect of individual error in determining the APACHE II score is trivial in comparison to the underlying trend of the group as a whole. In this guise the tool has been used to predict the outcome of classes of patients as varied as those with acute pancreatitis to patients with acute community acquired pneumonia. The original authors of the APACHE II system emphasized that although the APACHE II score was highly correlated with risk of death, an individual score could not be translated into a specific risk of death without taking into account the underlying diagnosis [1]. Thus in a large group of patients, all with an APACHE II score of 22 and the same clinical diagnosis, for example pneumonia, the risk of death would be very similar. However, the risk of death would not necessarily be the same as another group of patients also with an APACHE II score of 22 who had a different underlying diagnosis, for example, ascending cholangitis. A specific example of this was cited in the original paper [1]. Despite the different weighting given to the presence of chronic health conditions in the emergency surgical patient, there was still a substantial difference in observed mortality between medical and surgical patients. APACHE II seems to perform less well in surgical patients [7]. These cited limitations clearly show that applying a single APACHE II score cut-off to determine high risk of death to all classes of patients is less than optimal.

The second type of review has focused on the performance of small groups of individuals in achieving accuracy and reproducibility of the actual APACHE II. As Rowley and Fielding [6] have shown, inter-rater reliability alone is insufficient grounds for confidence in the accuracy of real-world APACHE II scores. In studies where the accuracy of an individual APACHE II determination is the main focus of attention, the number of cases that can be studied is necessarily limited given the intensive effort required to determine what the 'gold standard' value really is. We are not aware of any studies that attempt to examine the consequences of random or systematic errors on the performances of the APACHE II predictive model.

Although the absolute rate of erroneous APACHE II score determination that we have reported appears to be higher than that reported either by Polderman *et al*. [4] or Chen *et al*. [5], this may be largely attributable to the greater severity of physiologic derangements used in our simulations. Thus, the mean and median APACHE II score in Polderman's repeat scoring exercise was 14.3 (± 4.4) and 13.9 before rigorous training and 18.9 (± 2.4) and 16.2 after training. They do not provide adjudicated or 'gold standard' values for the individual patients they studied, so that strict comparisons of accuracy as opposed to inter-rater agreement cannot be made. The simulations we used had APACHE II scores of 19, 22 and 44. The opportunity for error rises almost geometrically with the number of deranged physiologic variables, which likely explains the higher standard deviation we observed in the simulations with the higher APACHE II scores. The overall intra-class correlation which we report (0.51) lies between the worst individual component value reported by Chen *et al*. [5] (for Glasgow Coma Score at 0.315) and the best (for age at 0.976). We did not perform intra-class correlations for individual elements of the APACHE II score. Despite the intrinsic variability noted by Chen *et al*. [5], when groups of patients were compared (as was intended by the designers of the original tool) the correlation was excellent.

The inter-rater reliability noted in this investigation (0.51) can, at best, be described as only fair. From a research perspective this underscores the potential bias in any critical care study relying on the APACHE II score either for entry into a trial or for analysis of baseline severity of illness. Moreover, if in the future novel therapies are to be targeted based on such a criterion, many patients eligible for a therapy may be excluded whereas others may be treated inappropriately. That we studied only trained researchers reinforces this point, as it seems reasonable to conclude that less specifically trained personnel or clinicians will likely make more errors in the computation of the APACHE II score. Future research in critical care might include multiple measures of severity of illness to address this limitation

Recently, it has been suggested that the APACHE II score may be a useful tool to determine if a patient has a sufficient risk of death to warrant treatment with drotrecogin alfa (activated). For a population of severe sepsis patients enrolled in the PROWESS trial [8], the APACHE II score was the strongest indicator for distinguishing patients with a response to the drug from the group that did not show a positive response [8]. Explicitly, the current US package insert for drotrecogin alfa (activated) proposes an APACHE II score of 25 or greater as a way to determine if a patient is at high risk of death [9]. Even if it is assumed that APACHE II methodology is perfect for resolving the arbitrary distinction between high risk of death and not at high risk of death, the error rate in determining the APACHE II score, which others have reported and which we have confirmed, will ensure that significant numbers of patients will be misclassified (i.e. they will be assigned to one side of a 25 point threshold when their true score lies on the other). There is a fundamental practical difference between using a scoring system such as APACHE II for examining likelihood of death, and using it to determine if a severe sepsis patient lies above or below an arbitrary threshold. In any given intensive care unit population; the majority of survivors are clustered at the low end of the APACHE II score range. Deaths are concentrated at the high end. If, in a population of patients, the observed mortality is plotted against APACHE II score, at the low end of the range the curve is quite flat. A change of score from 4 to 8 makes little difference to mortality; the vast majority still survive. Likewise, at the upper end of the range, above a score of about 40, most patients die, and increasing the score by two or three points changes the mortality little. In the mid-range of the curve, however, the mortality versus APACHE II score is very steep. A change of one or two points makes a large difference in the observed change in mortality. Thus, when using a cut-off point that happens to lie in the steepest region of the curve, the significance of scoring errors is maximized. The closer a patient's true APACHE II score approaches the cut-off point of 25, the higher the misclassification rate (this trend is illustrated in Fig. 2). Unfortunately, a cut-off value of 25 sits uncomfortably close to the median APACHE II score of 22, seen in severe sepsis patients admitted to intensive care units included in the PROGRESS registry (Fig. 3). The chance of misclassification for a patient lying within the inter-quartile range (17 to 28) is estimated to be as high as 38%. This set of patients represents the population of severe sepsis patients admitted to the intensive care unit for whom the outcome is most in doubt. Because the APACHE II scoring error rate estimates are based on a normal distribution around the true APACHE II score, these misclassification rates are conservative in nature, as the maximum misclassification rate can only be 50%. The real world distributions of scoring errors, such as seen in the scenario with APACHE II score of 22, suggest that occasionally the misclassification rate can exceed 50%. If such a score is to be used in a medical decision making process, the likely error rate should be clearly understood, and serious attention should be paid to maximizing the expertise and accuracy of those responsible for the scoring process.

## Conclusion

It is far more likely that an individual patient will be scored incorrectly than correctly, even by a group of individuals trained in scoring and chart abstraction. Even the scenario with an adjudicated APACHE II score that placed it many points distant from an arbitrary cut-off point of 25 was misclassified at an unacceptably high rate. Observed misclassification rate for the scenario with an adjudicated score within 3 points of the cut-off was over 50%. Integrating our study of APACHE II score errors with the published literature leads us to conclude that the APACHE II is an inappropriate sole tool for resource allocation decisions for individual patients.

## Key messages

• 	There are typically errors in execution of a complex scoring scheme such as APACHE II.

• 	These errors do not have a significant effect when applied to patient populations of a sufficient size.

• 	If a cut-off APACHE II score in the middle range of critically ill patients is used for making decisions about individual patients, an error rate that may be considered acceptable for use with sufficiently large patient populations will produce a very high rate of misclassification in those individuals so classified.

## Abbreviations

APACHE II = Acute Physiology and Chronic Health Evaluation II.

## Competing interests

FVMcLB, MS, NA, BB, RLQ and HL are full-time employees and shareholders of Eli Lilly and Company. AFS has been a paid consultant and speaker for Eli Lilly and Company.

## Authors' contributions

All the authors have contributed to the composition, revision and review of the manuscript and have read and approved the final version. In addition, FVMcLB and MS conceived of the idea for this manuscript, BB performed the statistical analysis, RLQ edited the document and BB participated in obtaining the original PROWESS data.

## Figures and Tables

**Table 1:** Error rates of combined case study data for each component of the APACHE II score

Acute physiologic score (A)	Error rates
Temperature (rectal/core)	48%
Mean arterial pressure	59%
Heart rate (ventricular response)	46%
Respiratory rate (non-ventilated or ventilated)	45%
Oxygenation	52%
Arterial pH	38%
Serum sodium	29%
Serum potassium	26%
Serum creatinine	43%
Hematocrit	33%
White blood count	49%
Glasgow Coma Scale	69%
Total acute physiology score (A)	87%
Age points (B)	10%
Chronic health points (C)	34%
Total APACHE II score (A+B+C)	86%
